# Antimicrobial elution patterns correlate with antibacterial and antifungal activities of three antimicrobial central venous catheters in serum

**DOI:** 10.1128/aac.01920-25

**Published:** 2026-04-20

**Authors:** Issam I. Raad, Y-Lan Truong, Bahgat Gerges, Ying Jiang, Ray Hachem, Peter Lamie, Anne-Marie Chaftari, Joel Rosenblatt

**Affiliations:** 1 Department of Infectious Diseases, Infection Control and Employee Health, The University of Texas MD Anderson Cancer Center 4002, Houston, Texas, USA; 2 Department of Hospital Medicine, The University of Texas MD Anderson Cancer Center 4002, Houston, Texas, USA; University Children's Hospital Münster, Münster, Germany

**Keywords:** central venous catheter (CVC), CVC-related bloodstream infection (CRBSI), microbial colonization, antifungal, antibacterial

## Abstract

Antimicrobial central venous catheters (CVCs) help prevent CVC-related bloodstream infection (CRBSI), and their effectiveness depends on sustaining broad antimicrobial activity in serum. A new CVC containing minocycline, rifampin, and chlorhexidine (MRC) was recently approved, complementing existing minocycline-rifampin (MR) and chlorhexidine-silver-sulfadiazine (CSiSz) CVCs. This study compared the elution profiles of minocycline, rifampin, and chlorhexidine from these three CVC types in serum and assessed resulting antimicrobial durability against CRBSI pathogens. MRCs were prepared by impregnation with minocycline and rifampin, followed by sequential chlorhexidine coating; commercial MR and CSiSz CVCs served as comparators. CVCs were immersed in plasma for 24 h, and then in serum for 3 weeks. At baseline and weekly intervals, CVC segments were analyzed for antimicrobial content and challenged with clinical CRBSI isolates. MRC retained higher minocycline and rifampin levels than MR and higher chlorhexidine levels than CSiSz after 3 weeks in serum. Correspondingly, MRC demonstrated superior inhibition of gram-negative colonization (*Klebsiella pneumoniae*, *Escherichia coli*, *Acinetobacter baumannii*) and *Enterococcus faecalis* at week 3 (*P* ≤ 0.028). They also showed greater activity against *Candida* species (spp.) (*C. albicans*, *C. glabrata*) at weeks 2 and 3 (*P* ≤ 0.005). MR and MRC CVCs completely prevented methicillin-resistant *Staphylococcus aureus* and *S. epidermidis* colonization for 3 weeks, outperforming CSiSz (*P* = 0.003). In conclusion, slow elution from MRC CVCs resulted in significantly enhanced antimicrobial durability against gram-negative bacteria, *E. faecalis*, and *Candida* spp. MR and MRC CVCs provided equivalent, sustained protection against *Staphylococcal* colonization, both surpassing CSiSz CVCs.

## INTRODUCTION

The use of central venous catheters (CVCs) in vascular therapy administration continues to evolve and expand. Traditionally, CVCs were used for administration of medications, nutrition, hydration, blood products, hemodialysis, and for diagnostic sampling or measurements when rapid dilution or infusion in vessels with high flow rates was required (1). With the recent expansion of the use of novel immunotherapies, such as chimeric antigen receptor (CAR) T-cell therapy and of hematopoietic cell transplantation (HCT), longer dwell times for CVCs have been required. The expanded use of CVCs comes with potential drawbacks, however. One is the risk of CVC-related bloodstream infections (CRBSIs), which increases with the CVC dwell time (2). Preventing CRBSI is critical to the success of immunotherapies because patients are immunocompromised and immunosuppressed during therapy. CRBSIs can produce serious morbidities resulting in high mortality rates for such patients (3).

Procedural bundles have been introduced to reduce the risk of CRBSIs (4). Another major line of defense against these infections is the use of antimicrobial CVCs that prevent colonization and proliferation of microbes on CVC surfaces while indwelling intravenously. The U.S. Food and Drug Administration (FDA) approved the use of antimicrobial CVCs over the past two decades to reduce the risk of CRBSIs (5). One leading antimicrobial CVC has a coating of chlorhexidine, silver, and the antibiotic sulfadiazine (CSiSz CVC), whereas another is coated with the antibiotics minocycline and rifampin (MR CVC) (6). Silver-coated CVCs were commercially available but largely had short durations of antimicrobial efficacy and were used less often (6). Several detailed reviews of the mechanisms of action and limitations of these silver-coated antimicrobial CVCs have been published (6).

The antimicrobial agents applied to the most widely used CSiSz and MR CVCs have not been updated over the past two decades (5, 6). During this interval, whereas new therapies have evolved, pathogenic threats have also evolved. From 2006–2007 to 2015–2017, the prevalence of gram-positive, coagulase-negative staphylococci (CoNS) decreased from 34.1 to 11.5% of CRBSIs in U.S. hospitals (7, 8). Simultaneously, the prevalence of *Candida* spp. CRBSIs increased from 11.8 to 17.5%; that of Gram-negative *Escherichia coli* CRBSIs increased from 2.70 to 7.25%; and that of gram-negative *Klebsiella pneumoniae* CRBSIs increased from 4.9 to 8.5% (7, 8). Hence, whereas a notable shift in the prevalence of pathogens from gram-positive to -negative bacteria and *Candida* spp. occurred when the CSiSz and MR CVCs were originally developed and introduced, the antimicrobial protection provided by antimicrobial CVCs has not changed appreciably as both pathogenic threats and CVC usage durations have evolved (7, 8). In the present study, we tested *in vitro* the antimicrobial activity of CSiSz and MR CVCs and a recently FDA-approved CVC with an updated antimicrobial coating of minocycline, rifampin, and chlorhexidine (MRC CVC) against CRBSI pathogens in a clinically relevant *in vitro* model of microbial colonization (9). Several studies on antimicrobial CVC (using MR and CSiSz CVC) have established the correlation between quantitative burden of catheter colonization and the subsequent incidence of catheter-related bloodstream infections (CRBSI), demonstrating quantitatively that catheter colonization is the prelude to CRBSI (10, 11). Hence, since antimicrobial CVCs quantitatively decrease the risk of catheter colonization, they have been shown to decrease the risk of CRBSI. This is why in this study we focused on the efficacy of antimicrobial catheters in quantitatively decreasing the risk of catheter colonization as a predictor of decreasing CRBSI.

Because CVC dwell times have evolved over time, we also assessed the durability of protection against various bacterial and *Candida* spp. CRBSI pathogens as well as that of retention of the bioactive agents by the CVCs during simulated elution. We measured retention of the bioactive antimicrobial agents using high-performance liquid chromatography and at the same simulated dwell time points when antimicrobial activity was assessed to determine whether the underlying elution profiles of the different CVCs affected antimicrobial durability against the broad spectrum of tested CRBSI pathogens.

## MATERIALS AND METHODS

### CVC preparation

Sterile triple-lumen 7 French CVCs were used for this study. CSiSz CVCs (Arrowgard Blue Plus) were purchased from Teleflex Medical (Morrisville, NC, USA), while MR and MRC CVCs were provided by Cook Medical (Bloomington, IN, USA), as were nonantimicrobial control polyurethane CVCs. Sterile CVCs were aseptically cut under a biosafety cabinet into 1-cm segments. The segments were protected from light and stored at room temperature until use. Commercially available CVCs were used prior to expiration. MRC CVCs were used within 6 months of manufacture.

### Conditioning and elution of CVCs to simulate indwelling *in vivo*

All CVCs were tested at four time points: baseline, 1, 2, and 3 weeks. At each time point, six replicates of 1-cm segments of each CVC type (nonantimicrobial control polyurethane CVCs, CSiSz CVCs, MR CVCs, and MRC CVCs) were tested against each organism and evaluated. For baseline testing, CVC segments were soaked in human plasma (Innovative Research, Novi, MI, USA) for 24 h, and then exposed to microbial challenge inocula as described below. For durability testing (1, 2, and 3 weeks), CVC segments were soaked in plasma for 24 h, and then soaked in newborn calf serum (Thermo Fisher Scientific, Waltham, MA, USA) until microbially challenged at the respective time points. Serum was refreshed weekly throughout antimicrobial durability testing. All microbiologic testing and manipulations were aseptically conducted using a biosafety cabinet.

### Microbial challenge at each time point

Microbial challenge organisms used to test the CVCs were clinical bloodstream infection pathogens obtained from patients with cancer at MD Anderson as representatives of prevalent gram-positive and -negative and fungal CRBSI pathogens. The following pathogens were used for testing: *E. coli* (strain MB 2839), *K. pneumoniae* (strain MB 4357), *Acinetobacter baumannii* (strain MB 1000), methicillin-resistant *Staphylococcus aureus* (MRSA; strain MDA 120), *S. epidermidis* (strain MB 8839), *Enterococcus faecalis* (strain MB 7178), *C. albicans* (strain MDA 177), and *C. glabrata* (strain MDA 2249A). Briefly, challenge inocula were prepared by removing stock cultures stored at −80°C, thawing them at room temperature, culturing them onto trypticase soy agar and 5% sheep blood (blood agar; for bacterial pathogens) and Sabouraud dextrose agar (catalog #R01766; Thermo Fisher Scientific, Inc., Waltham, MA, USA; for *Candida* spp. pathogens), and incubating them at 37°C for 24 h. Once grown, uniform colonies of each test organism were picked up by a sterile loop and inoculated into Mueller Hinton broth (MHB) (BBL Mueller Hinton II Broth [cation adjusted], catalog #B12322; Thermo Fisher Scientific, Inc., Waltham, MA, USA) for bacterial pathogens and yeast peptone dextrose (YPD) (catalog #DF0428175; Thermo Fisher Scientific, Inc., Waltham, MA, USA) for *Candida* spp. Pathogens were incubated for 1–2 h at 37°C. The inoculum was then diluted to a 0.5 McFarland standard (~1.5 × 10^8^ CFU/mL for bacteria and ~1 × 10^6^ CFU/mL for *Candida* spp.) using a turbidity meter, and then further diluted in MHB (bacterial pathogens) or YPD (*Candida* spp. pathogens) to the target inoculum concentration (10^5^ CFU/mL) for each experiment.

Microbial challenge was performed at each time point with six replicates by placing a 1 cm segment of each tested CVC separately into a single well of a 24-well tissue culture plate and pipetting 1 mL of each target inoculum into the wells of each CVC segment. If CVC segments were floating in the inoculum, a sterile stick was used to push them into the inoculum to make sure they were fully submerged. Segments were incubated at 37°C and shaken at 100 rpm for 24 h to colonize the CVC segments. After colonization, liquid broth was gently removed from the well of the tissue culture plate without disturbing the segments. Segments were subsequently washed in isotonic sterile saline, shaking at 100 rpm at 37°C for 30 min to remove any organisms that were not adherent to the biofilm. After washing in isotonic sterile saline, the segments were then placed in 1 mL of Dey-Engley neutralizing broth (catalog #DF0819-17-2; Thermo Fisher Scientific, Inc., Waltham, MA, USA) and sonicated for 15 min to disrupt attached microbes and assess whether any viable organisms remained on the CVC segments. The sonicate was vortexed for 30 s and serially diluted for quantitative culture. Viable recovered organisms were quantified by plating 100 mL of serial dilutions on blood agar (for bacterial isolates) or Sabouraud dextrose agar (for *Candida* spp. isolates) and incubated for 24–48 h at 37°C. After incubation, the total viable colonies on the plates were enumerated.

### High-performance liquid chromatography analysis of the minocycline, rifampin, and chlorhexidine content of CVC segments

Following microbial challenge, the CVC segments were harvested from the culture plate and stored for chemical analysis of the minocycline, rifampin, and chlorhexidine content remaining in them. Segments from CVCs that were not conditioned were used to measure the initial minocycline, rifampin, and chlorhexidine levels. Minocycline, rifampin, and chlorhexidine were fully extracted from 12 CVC segments via incubation overnight in methanol. Solutions were diluted to an assay range of 2–20 mg/mL and injected onto a high-performance liquid chromatography system (Inotiv, West Lafayette, IN, USA) with a Luna C18 column (Phenomenex, Torrance, CA, USA). A methanol gradient was employed with 20 mM sodium phosphate buffer (pH 3) as the mobile phase. Analyte detection was by ultraviolet absorbance at 260 nm. The run time for minocycline, rifampin, and chlorhexidine was 17.5 min, with peaks eluting at 8.3, 11.9, and 14.5 min, respectively. Minocycline, rifampin, and chlorhexidine reference standards (LGC Standards, Manchester, NH, USA) were used to specify peak elution times.

### Statistical analysis

Continuous variables with a normal distribution were summarized as means, whereas those with a nonnormal distribution were summarized as medians and ranges. Differences in antimicrobial activity, indicated by colony-forming unit (CFU) counts between CVC types, were compared using the Wilcoxon rank-sum test due to non-normal data distribution. Additionally, the log₁₀ reduction in CFU counts for each CVC type relative to the control CVCs was calculated. All statistical tests were two-sided, and *P*-values ≤ 0.05 were considered significant. Data analyses were performed using SAS software (version 9.4; SAS Institute, Inc., Cary, NC, USA).

## RESULTS

**Fig 1:**
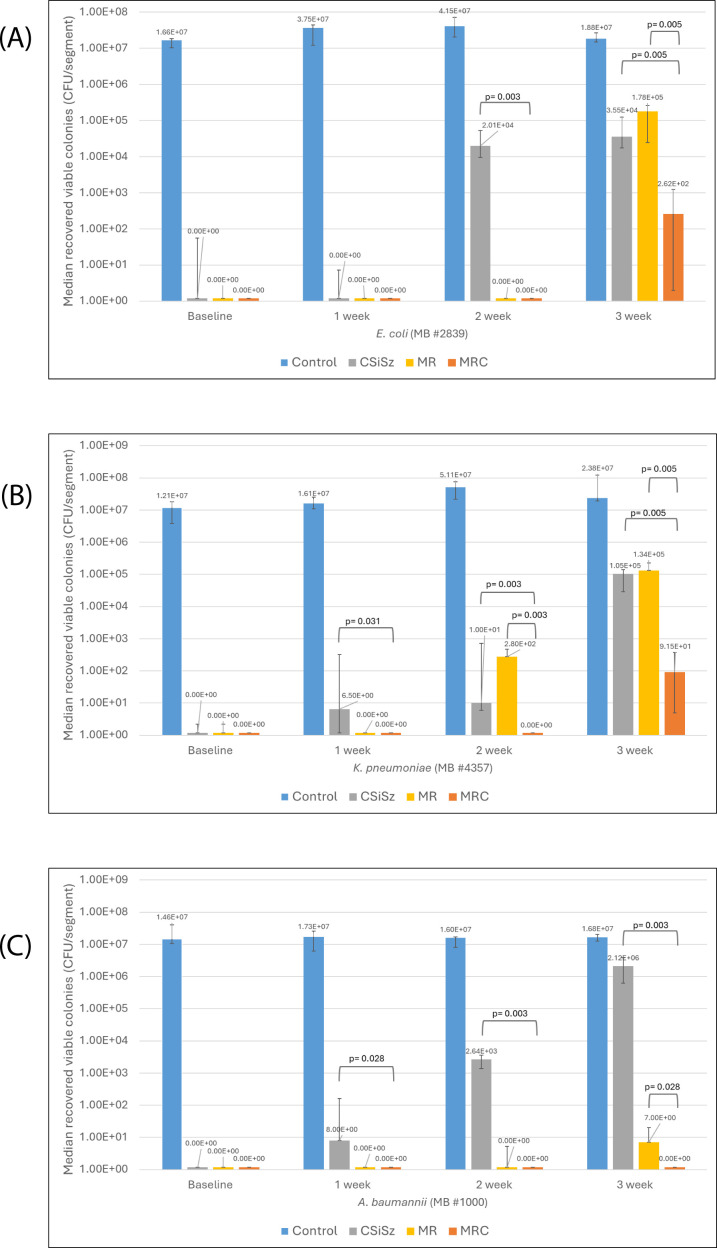
** **Median numbers of colonies of (**A**) *Escherichia coli*, (**B**) *Klebsiella pneumoniae*, and (**C**) *Acinetobacter baumannii* recovered at baseline and over 3 weeks from the minocycline-rifampin-chlorhexidine (MRC) central venous catheters (CVCs) compared with the control, chlorhexidine-silver-sulfadiazine (CSiSz), and minocycline-rifampin (MR) CVCs. Graph is presented as median values with range bars for a total of six replicates. Time points when significant (*P* ≤ 0.05) differences in the number of colony-forming unit (CFU)/segment between the CSiSz and MRC CVCs and between the MR and MRC CVCs were present are indicated by brackets with calculated *P*-values. (**A**) The differences in antimicrobial activity between CSiSz and MRC CVCs were significant at weeks 2 (*P* = 0.003) and 3 (*P* = 0.005). The differences in antimicrobial activity between MR and MRC CVCs were significant at week 3 (*P* = 0.005). (**B**) The differences in antimicrobial activity between CSiSz and MRC CVCs were significant at weeks 1 ( *P* = 0.031), 2 (*P* = 0.003), and 3 (*P* = 0.005). The differences in antimicrobial activity between MR and MRC CVCs were significant at weeks 2 (*P* = 0.003) and 3 (*P* = 0.005). (**C**) The differences in antimicrobial activity between CSiSz and MRC CVCs were significant at weeks 1 ( *P*= 0.028), 2 (*P* = 0.003), and 3 (*P* = 0.003). The differences in antimicrobial activity between MR and MRC CVCs were significant at week 3 (*P* = 0.028).

Breakthrough colonization of *E. coli* occurred at week 2 for the CSiSz CVC but not until week 3 for the MR and MRC CVCs (Fig. 1A). Furthermore, at 3 weeks, the MRC CVC had more significant *E. coli* colonization reduction than did the MR and CSiSz CVCs (*P* = 0.005), with about a 5 log_10_ reduction when compared with control uncoated CVCs. For *K. pneumoniae*, slight breakthrough colonization occurred at weeks 1 and 2 for the CSiSz CVC, with more substantial breakthrough colonization at week 3 (Fig. 1B). Like with *E. coli*, at 3 weeks, the MRC CVC had more significant *K. pneumoniae* colonization reduction than did the MR and CSiSz CVCs (*P* = 0.005), with more than 5 log_10_ reduction when compared with control uncoated CVCs. For *A. baumannii*, initial breakthrough colonization occurred at week 1 for the CSiSz CVC, with progressively more breakthrough colonization at weeks 2 and 3 (Fig. 1C). We observed no breakthrough *A. baumannii* colonization for the MRC CVC but more significant breakthrough colonization for the MR CVC at 3 weeks (*P* = 0.028).

**Fig 2:**
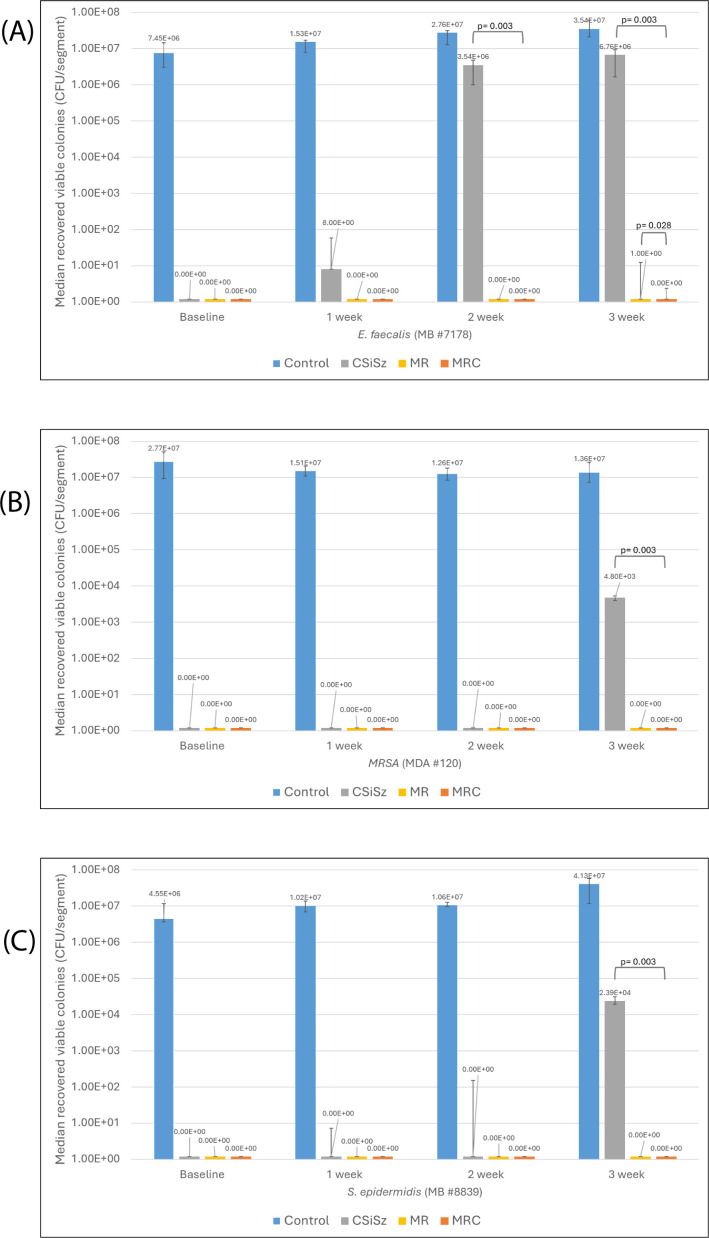
** **Median numbers of colonies of (**A**) *Enterococcus faecalis*, (**B**) methicillin-resistant *Staphylococcus aureus* (MRSA), and (**C**) *S. epidermidis* recovered at baseline and over 3 weeks from the minocycline-rifampin-chlorhexidine (MRC) central venous catheters (CVCs) compared with the control, chlorhexidine-silver-sulfadiazine (CSiSz), and minocycline-rifampin (MR) CVCs. Graph is presented as median values with range bars for a total of six replicates. Time points when significant (*P* ≤ 0.05) differences in the numbers of colony-forming unit (CFU)/segment between CSiSz and MRC CVCs and between MR and MRC CVCs were present are indicated by brackets with calculated *P*-values. (**A**) The differences in antimicrobial activity between CSiSz and MRC CVCs were significant at weeks 2 (*P* = 0.003) and 3 (*P* = 0.003). The differences in antimicrobial activity between MR and MRC CVCs were significant at week 3 (*P* = 0.028). (**B**) The differences in antimicrobial activity between CSiSz and MRC CVCs were significant at week 3 (*P* = 0.003). (**C**) The differences in antimicrobial activity between CSiSz and MRC CVCs were significant at week 3 (*P* = 0.003).

Regarding *E. faecalis*, we saw breakthrough colonization with the CSiSz CVC at week 1 and overwhelming breakthrough colonization for this CVC at weeks 2 and 3 (Fig. 2A). In contrast, the MRC CVC was completely resistant to *E. faecalis* colonization for 3 weeks, and the MR CVC had significant breakthrough colonization at 3 weeks (*P* = 0.028). In addition, all three antimicrobial CVCs were effective against MRSA for 2 weeks. At week 3, the CSiSz CVC had breakthrough MRSA colonization, whereas the MR and MRC CVCs had no colonization (Fig. 2B). We observed similar results for *S. epidermidis* (*P* = 0.003) (Fig. 2C).

**Fig 3:**
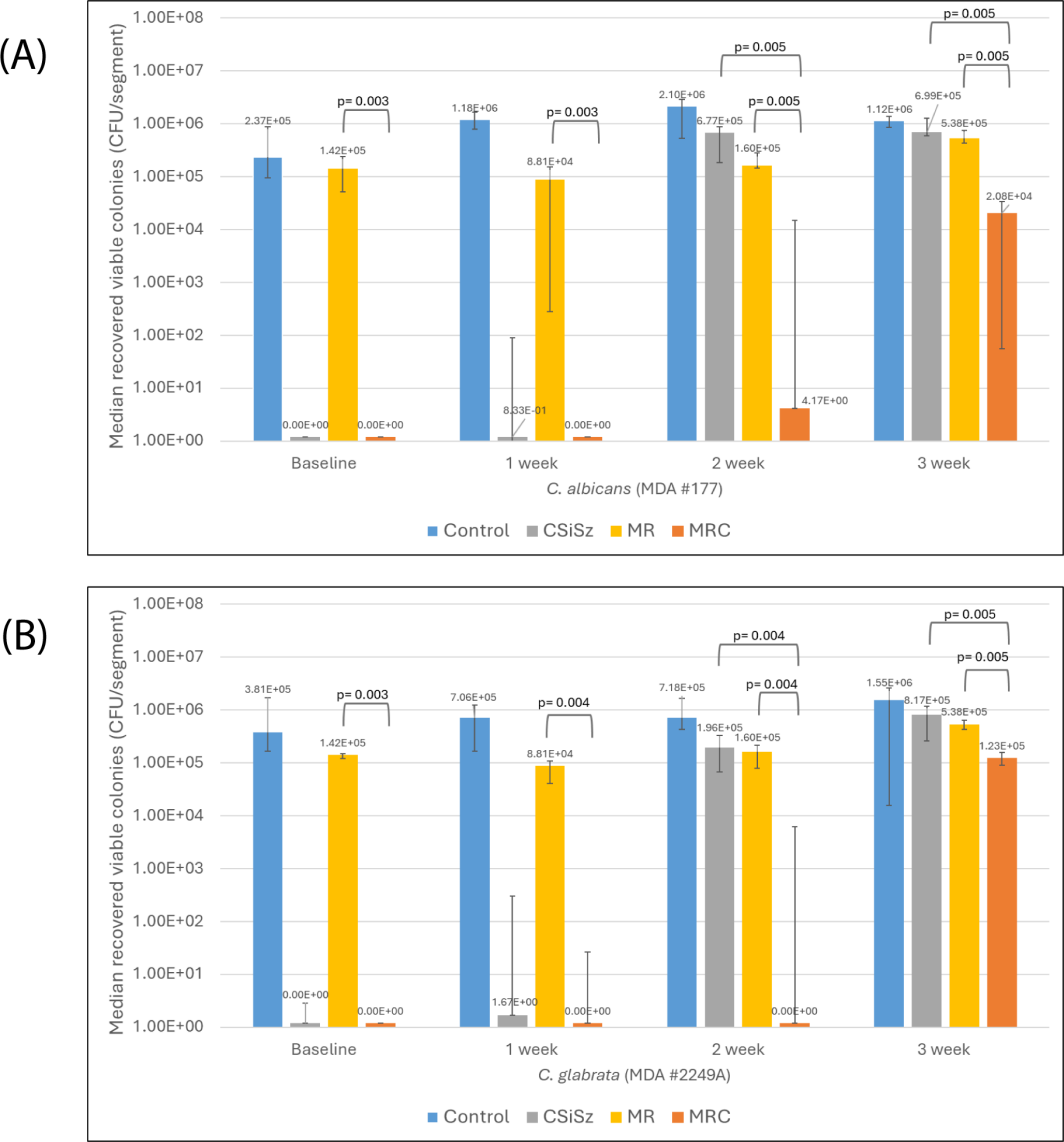
Median numbers of colonies of (**A**) *Candida albicans* and (**B**) *C. glabrata* recovered at baseline and over 3 weeks from the minocycline-rifampin-chlorhexidine (MRC) central venous catheters (CVCs) compared to control, chlorhexidine–silver–sulfadiazine (CSiSz), and minocycline-rifampin (MR) CVCs. Graph is presented as median values with range bars for a total of six replicates. Time points when significant (*P* < 0.05) differences in the number of colony-forming unit (CFU)/segment between the CSiSz and MRC CVCs and between MR and MRC CVCs are indicated by brackets with calculated *P*-values. (**A**) The differences in antimicrobial activity between CSiSz and MRC CVCs were significant at weeks 2 (*P* = 0.005) and 3 (*P* = 0.005). The differences in antimicrobial activity between MR and MRC CVCs were significant at baseline (*P* = 0.003), week 1 (*P* = 0.003), week 2 (*P* = 0.005), and week 3 (*P* = 0.005). (**B**) The differences in antimicrobial activity between CSiSz and MRC CVCs were significant at weeks 2 (*P* = 0.004) and 3 (*P* = 0.005). The differences in antimicrobial activity between MR and MRC catheters were significant at baseline (*P* = 0.003), week 2 (*P* = 0.004), and week 3 (*P* = 0.005).

Furthermore, the MR CVC was ineffective at preventing colonization by both *Candida* spp. (Fig. 3A and B). Substantial colonization of both species occurred at week 2 for the CSiSz CVC but not for the MRC CVC (*P* = 0.005). The MRC CVC was significantly more effective in reducing colonization by *Candida* spp. at weeks 2 and 3 than were the MR and CSiSz CVCs (*P* = 0.005).

Table 1 lists the mean minocycline, rifampin, and chlorhexidine concentrations remaining in the tested CVC segments at each time point. Day 0 reflects the mean initial loading of CVC segments prior to elution. The initial loading of chlorhexidine was more than 20% greater in the CSiSz CVC than in the MRC CVC. Also, the initial minocycline loading was about 10% greater in the MRC CVC than in the MR CVC. However, the initial rifampin loading in the MR CVC was more than 20% greater than that in the MRC CVC.

**TABLE 1:** Mean chlorhexidine, minocycline, and rifampin (micrograms/cm) recovered from chlorhexidine-silver-sulfadiazine (CSiSz), minocycline-rifampin (MR), and minocycline-rifampin-chlorhexidine (MRC) central venous catheter (CVC) segments at each time point following elution^*a*^

Mean value (mg/cm)		
Chlorhexidine		
Day	CSiSz CVC	MRC CVC
0	375	296
1	73	202
7	52	136
14	53	107
21	52	86
Minocycline		
Day	MR CVC	MRC CVC
0	361	405
1	284	197
7	60	83
14	13	44
21	8	23
Rifampin		
Day	MR CVC	MRC CVC
0	509	404
1	445	281
7	295	197
14	175	165
21	102	126

^*a*^ Day 0 is the time of initial loading prior to elution.

The amounts of the three antimicrobial agents released between time points can be estimated according to the difference in mean content remaining in a CVC segment between one time point and the previous one. We found no change in the chlorhexidine amount for the CSiSz CVC from days 14 to 21, as the difference was less than 1% of the initial loading. This reflects that significant elution of chlorhexidine did not occur following day 7 for the CSiSz CVC (although non-eluting chlorhexidine remained in the CVC). Chlorhexidine continued to elute from the MRC CVC through day 21. The MR and MRC CVCs appeared to continue eluting minocycline and rifampin in parallel manners over 3 weeks. The addition of eluting chlorhexidine from the MRC CVC is a more consequential difference underlying their different antimicrobial durabilities.

## DISCUSSION

Over the past decade, central venous catheters (CVCs) in high-risk critically ill populations have remained in place for increasingly prolonged durations, often several weeks. Longer dwell times of short-term CVCs are independently associated with higher rates of CVC-related bloodstream infections (CRBSIs), as demonstrated in multiple ICU cohort studies (12, 13). A large post hoc analysis of 15,036 catheters in 24 ICUs showed that infection risk increased significantly after 10 days *in situ*, particularly for CVCs placed at non-subclavian sites (14). Other studies have demonstrated a time-dependent relationship between dwell time and infection risk, with prolonged hospitalization prior to insertion and extended catheter duration identified as key risk factors (15, 16). These findings provided the rationale for the present study, which evaluated antimicrobial durability over a 3-week period using rigorous *in vitro* colonization testing of three FDA-approved antimicrobial CVCs. Because polyurethane CVC surfaces rapidly become coated with plasma and serum proteins, antimicrobial elution profiles are critical determinants of microbial attachment during prolonged blood exposure (17).

Our data demonstrate that the slower elution profile of the minocycline-rifampin-chlorhexidine (MRC) CVC resulted in higher combined antimicrobial agent levels in serum at 3 weeks compared with minocycline-rifampin (MR) and chlorhexidine-silver sulfadiazine (CSiSz) CVCs. This was associated with markedly greater antimicrobial durability than that of the MRC CVC against gram-negative bacteria, *E. faecalis*, and *Candida* spp. at week 3. Both MR and MRC CVCs completely prevented colonization by staphylococci throughout the 3-week period and demonstrated greater durability than the CSiSz CVC. These findings are consistent with prior studies showing that MRC catheters inhibit biofilm formation by multidrug-resistant gram-negative organisms, vancomycin-resistant enterococci, MRSA, and *Candida* spp. after extended dwell times (18–20). The present study uniquely demonstrates that the superior antimicrobial durability of the MRC CVC correlates directly with the sustained, slow release of all three antimicrobial agents. This may be attributable to its initial impregnation with minocycline and rifampin, followed by sequential internal and external chlorhexidine coating, which likely delays elution and prolongs antimicrobial activity (19).

These findings have particular relevance for gram-negative CRBSIs, which have increased in frequency over recent decades and tend to occur several weeks after CVC insertion (7, 8, 21–23). Prior studies have shown that gram-negative CRBSIs are associated with longer dwell times and are increasingly multidrug resistant, particularly in patients with cancer and neutropenia (21–23). In the present study, breakthrough colonization of gram-negative pathogens occurred earliest with the CSiSz CVC, corresponding to its rapid chlorhexidine elution, whereas the MR and MRC CVCs prevented gram-negative colonization through week 2. At week 3, the MRC CVC was the only device achieving >4 log₁₀ reductions in *K. pneumoniae* and *E. coli* colonization, reflecting the combined and sustained elution of chlorhexidine, minocycline, and rifampin.

For gram-positive organisms, antibiotic retention-elution profiles correlated with complete prevention of staphylococcal colonization by the MR and MRC CVCs through 3 weeks, whereas breakthrough occurred with the CSiSz CVC at week 3. *E. faecalis* colonization occurred earlier with the CSiSz CVC, while the MRC CVC fully prevented colonization through week 3, a clinically relevant finding given the organism’s strong biofilm-forming capacity and its prominence as a cause of CRBSIs in long-term care settings (8, 24, 25)

The *Candida* spp. findings similarly correlated with chlorhexidine retention. The MRC CVC provided more durable protection than the CSiSz CVC, while the MR CVC was ineffective, consistent with the lack of antifungal activity of minocycline and rifampin. Given that *Candida* spp. account for a substantial proportion of ICU CRBSIs and are associated with high attributable mortality, these results are clinically significant (8, 26–29)

This study has limitations. *In vitro* findings require confirmation in prospective clinical trials evaluating both microbial colonization and CRBSI prevention over extended dwell times. Future studies should also assess clinical performance characteristics, including ease of insertion and thermogenic risk.

In conclusion, antimicrobial elution patterns over 3 weeks correlate closely with antimicrobial durability. At week 3, the MRC CVC demonstrated superior activity against Gram-negative bacteria, *E. faecalis*, and *Candida* spp. compared with MR and CSiSz CVCs, while MR and MRC CVCs showed equivalent and superior activity against staphylococci. Prospective clinical trials are needed to assess performance beyond 3 weeks.
